# CHANGES IN TECH USE PREDICTING SOCIAL WELL-BEING FOR INTERGENERATIONAL PROGRAM PARTICIPANTS

**DOI:** 10.1093/geroni/igad104.1719

**Published:** 2023-12-21

**Authors:** Cindy Tsotsoros, Skye Leedahl, Melanie Brasher, Alexandria Capolino, Josie Santilli

**Affiliations:** University of Rhode Island, Kingston, Rhode Island, United States; University of Rhode Island, Kingston, Rhode Island, United States; University of Rhode Island, Kingston, Rhode Island, United States; University of Rhode Island, Kingston, Rhode Island, United States; University of Rhode Island, Kingston, Rhode Island, United States

## Abstract

Previous research has shown that technology-based interventions show promise for reducing social isolation and loneliness. Less research has examined quality of life outcomes for these programs or interconnectedness of these outcomes when examining pre/post-change. This study examined the connections between technology use and knowledge with loneliness, social isolation, and quality of life before and after participation in the program. The final sample (N=324) was rather diverse regarding work and living status (for example, 67.5% retired; 67.8% lived alone; 70.1% had less than $30,000 income; 40.2% had a high school education or less). Using structural equation modeling, this study examined pre- and post-data to identify to what extent technology usage and knowledge (frequency of iPad use, digital competence) predicted an individual’s change in loneliness, social isolation, and quality of life. This final model showed excellent model fit (𝜒2(148, N=324)=247.01 p<.05 with CFI = .95, TLI = .93, RMSEA = .05, CMIN/df = 1.67). Within this model, findings showed that digital competence improvements predicted quality of life, social isolation, and loneliness (Ad. R. Square = 0.17); the frequency of iPad use influenced social isolation and quality of life (Adj. R Square = 0.29); and loneliness influenced quality of life and social isolation (Adj. R Square = 0.81). There are multiple possible mechanisms through which older participants’ lives can be impacted by intergenerational technology programs. Understanding the complex interplay of factors helps us understand the potential benefits of these programs on quality of life.

